# A case report of toxic epidermal necrolysis (TEN) in a patient with COVID-19 treated with hydroxychloroquine: are these two partners in crime?

**DOI:** 10.1186/s12948-020-00133-6

**Published:** 2020-10-06

**Authors:** Carlo Maria Rossi, Flavio Niccolò Beretta, Grazia Traverso, Sandro Mancarella, Davide Zenoni

**Affiliations:** 1 Dipartimento di Area Medica, U.O. Medicina Interna, ASST Nord Milano, Ospedale Edoardo Bassini, Via Massimo Gorki 50, 20092 Cinisello Balsamo (MI), Italy; 2 Scuola di Specializzazione in Farmacia Ospedaliera, Università degli Studi di Milano, Via L. Mangiagalli 25, 20133 Milan, MI Italy; 3 U.O.C. Farmacia Interna, ASST Nord Milano, Ospedale Edoardo Bassini, Via Massimo Gorki 50, 20092 Cinisello Balsamo (MI), Italy

**Keywords:** COVID 19, SARS-CoV- 2, Toxic Epidermal Necrolysis (TEN), Hydroxychloroquine, Severe Cutaneous Adverse Reactions (SCAR), Adverse Drug Reaction (ADR), Stevens–Johnson Syndrome (SJS), ALDEN Algorithm, SCORTEN Score, Pharmacovigilance

## Abstract

**Background:**

Stevens-Johnson Syndrome/Toxic Epidermal Necrolysis (SJS/TEN) is the most Serious Cutaneous Adverse Reaction (SCAR) often with a fatal outcome. Coronavirus Disease (COVID-19) is caused by Severe Acute Respiratory Syndrome–Coronavirus—2 (SARS-COV2) and is an emergent pandemic for which no cure exist at the moment. Several drugs have been tried often with scant clinical evidence and safety.

**Case presentation:**

Here we report the case of 78-years-old woman with cardiometabolic syndrome and COVID-19. A multidrug regimen including others hydroxychloroquine, antibiotics, dexamethasone and paracetamol, low-molecular-weight-heparin and potassium canrenoate was started. After almost 3 weeks, the patient started to display a violaceous rash initially involving the flexural folds atypical targetoid lesions and showing a very fast extension, blister formation and skin detachments of approximately 70% of the total body surface area and mucous membranes involvement consistent with toxic epidermal necrolysis (TEN). The ALDEN algorithm was calculated inserting all drugs given to the patient in the 28 days preceding the onset of the skin manifestations. The highest score retrieved was for hydroxychloroquine. Other less suspicious drugs were piperacillin/tazobactam, ceftriaxone and levofloxacin.

**Conclusions:**

To our knowledge, this is the first case of TEN in a patient suffering from COVID-19 probably associated with hydroxychloroquine. Given the activation of the immune system syndrome induced by the virus and the widespread off-label use of this drug, we suggest a careful monitoring of skin and mucous membranes in all COVID-19 positive patients treated with hydroxychloroquine in order to early detect early signs of toxicities.

## Background

Toxic Epidermal Necrolysis (TEN) is a rare Serious Cutaneous Adverse Reaction (SCAR). It displays an acute onset and is characterized by erythematous or violaceous patches, atypical targetoid lesions, bullae, erosions and skin detachment. It differs from Stevens-Johnson Syndrome (SJS) only in the percentage of skin involvement, which in TEN is greater than 30% of the body surface [1]. Etiopathogenetically, it results from the combination of drug and host genetic factors (such as drug metabolism and T cell clonotypes) resulting in a delayed-type hypersensitivity reaction, where drug or drug-peptide complexes are recognized by T cell receptors [2].

Coronavirus Disease (COVID-19) is caused by Severe Acute Respiratory Syndrome–Coronavirus—2 (SARS-CoV-2) and can be potentially fatal disease [3]. The most common symptoms at onset of COVID-19 illness are fever, cough, and fatigue, followed by dyspnea and diarrhea [4]. Lymphopenia, elevation of creatinine kinase (CK), lactate dehydrogenase (LDH) are also frequently observed. The lung involvement is frequently that of an interstitial pneumonia, COVID-19 later shows an over exuberant inflammatory response with a no correlation between viral load and the worsening of symptoms [5].

At the moment there is no effective cure for COVID-19, several drugs often in combination have been used, often with scant clinical evidence or conflicting results, with the aim of targeting the virus replication and/or the inflammatory process, such as anti-retrovirals, macrolides, hydroxychloroquine and monoclonal antibodies targeted on inflammatory cytokines [6–9]. There is limited data establishing their safety profile during SARS-CoV-2 infection.

## Case presentation

**Fig. 1:**
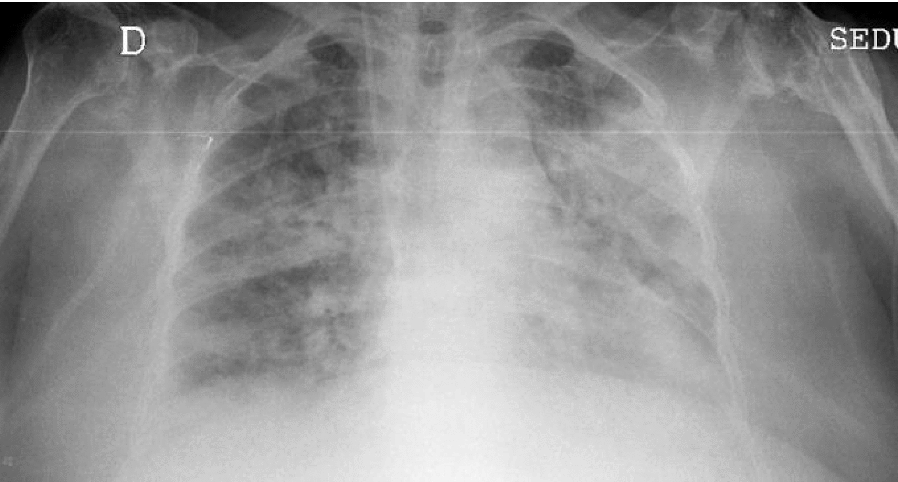
The X-ray shows bilateral and extensive interstitial infiltrates

Here we report the case of 78-year old woman with several comorbidities (hypertension, obesity, unstable angina, type 2 diabetes) admitted to our hospital for respiratory insufficiency with fever requiring non-invasive ventilation and a diagnosis of COVID-19 lung infection with bacterial superinfection was formulated given the radiological evidence of a bilateral interstitial pneumonia, the positivity of nasopharyngeal swab for SARS-CoV-2 and (Fig. 1). Lymphopenia (0.55 10^3^/µL [1.00–4.00]), elevation of CK (252 U/L [24–170]) and LDH (407 U/L [30–250]) were also observed.

A multidrug regimen with hydroxychloroquine 200 mg twice a day, sodium enoxaparin and dexamethasone were started. Besides, an antibiotic treatment with ceftriaxone for 1 day, followed by piperacillin/tazobactam for 4 days was given. The patient was initially treated in a sub-intensive care unit and after the favorable clinical evolution was transferred to our general medicine division, 18 days after the admission. At the arrival in our unit, the patient started to display a violaceous erythematous rash mainly involving the flexural folds. The patient was still taking hydroxychloroquine, along with levofloxacin (started the day before); other drugs were reported in Table 1.

**Table 1:** Calculation of the ALDEN score for each drug administrated during the hospitalization (chronic treatments were excluded) [10]

DRUG	ALDEN score	Causal link
Enoxaparin	−2	Very unlikely^a^
Oseltamivir	−2	Very unlikely
Ceftriaxone	+1	Unlikely
Potassium Canrenoate	−3	Very unlikely
Pantoprazole	−2	Very unlikely^a^
Hydroxychloroquine	+4	Possible
Piperacillin/Tazobactam	+1	Unlikely
Dexamethasone	0	Unlikely
Levofloxacin	+1	Unlikely
Paracetamol	−1	Very unlikely^a^

< 0: very unlikely

0–1: unlikely

2–3: possible

4–5: probable

≥ 6: very probable

^a^re-challenge without any adverse reactions. Score (−12; +10)

**Fig. 2:**
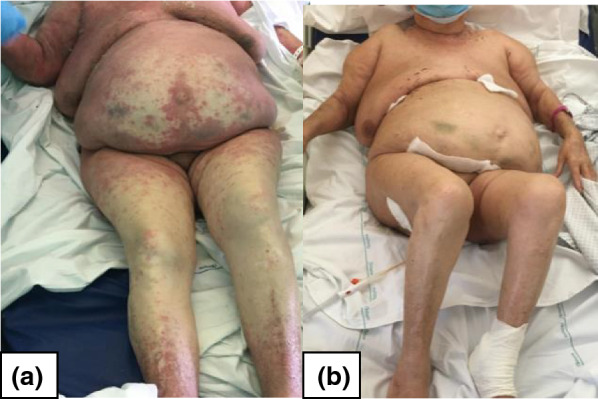
The violaceous rash extension with atypical targetoid elements (at day 3 from clinical onset) is depicted in **a**. **b** shows the complete resolution (after 6 weeks)

**Fig. 3:**
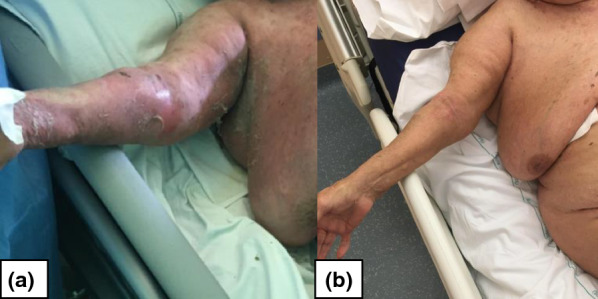
The extensive skin detachment is showed in **a** (at day 5) and its favorable evolution at 6 weeks in (**b)**

**Fig. 4:**
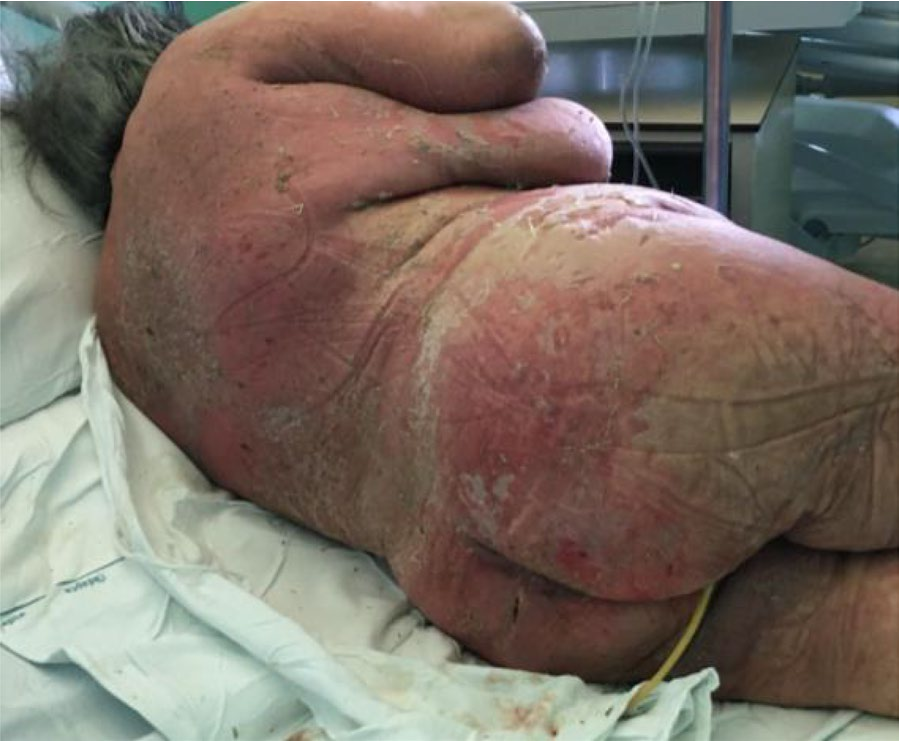
The figure shows the extensive disepithelialization with subcutaneous oozing and bleeding

The rash presented in the course of 3 days a rapid extension with the involvement of the whole trunk and buttocks, reaching approximately 70% of the total body surface area, with the appearance of atypical targetoid lesions and the formation of blisters, with subsequent skin detachment (Figs. 2, 3 and 4). Nikolsky’s sign was present. A severe desquamation of the buccal and nasal mucosa was also observed. The patient referred severe skin pain requiring morphine. Blood tests did not show eosinophilia or alterations of liver and renal function tests.

A clinical diagnosis of toxic epidermal necrolysis (TEN) also confirmed by the dermatologist was formulated. Methylprednisone 1 mg/kg, as an intravenous bolus regiment was administred along with intravenous immunoglobulin (IVIG) 1 mg/kg for 3 days. Then oral prednisone 1 mg/kg daily was given and subsequently tapered in 1 month.

To identify the culprit drug, the ALDEN algorithm was calculated (Table 1) [10] (Additional file 1). The highest score retrieved was for hydroxychloroquine, with a possible correlation (+4 score). Other less suspicious drugs were piperacillin/tazobactam, ceftriaxone and levofloxacin. All suspected drugs according to the ALDEN algorithm were promptly stopped (Additional file 2).

The patient was cared for by the wound care service of the hospital with topical therapy, a marked improvement in the skin conditions was observed progressively over a period of 6 weeks, until the complete resolution (Figs. 2 and 3). Paracetamol, pantoprazole, enoxaparin were tolerated after the reaction, ruling out their correlation with the skin reaction.

Due to the COVID-19 epidemic, no skin biopsies were technically feasible for logistical problems. However, the clinical manifestations were highly compelling for SJS/TEN. Other differential diagnosis included Drug-Reaction with Eosinophilia and Systemic Symptoms (DRESS), which was excluded given the absence of eosinophilia and internal organ involvement, Staphylococcal Scalded Skin Syndrome (SSSS), which is a pediatric form, and bullous dermatoses that were ruled out in our case given the minor mucous involvement and intense skin inflammation.

## Discussion

SJS/TEN is the most severe SCAR being associated with a high mortality rate [1]. It is a delayed reaction occurring after 4–28 days from drug exposure, therefore it is of paramount importance to acquire precise retrospective pharmacological information for a long period of time preceding the onset of skin manifestations.

STS/TEN has an approximate incidence of 1 or 2 cases/1,000,000 annually. It results from the clonal expansion of CD8 + cytotoxic T lymphocytes (CTLs) and Natural Killer cell (NK),which are major histo-compatibility complex (MHC)-restricted and induce epidermal apoptosis [1, 11, 12]. Recently, it has been shown that low molecular weight drugs can directly bind the T cell receptor (TCR) of T cells or the Human Leukocyte Antigen (HLA) of antigen-presenting cells [13–16].

Usually implicated drugs are allopurinol, anticonvulsant drugs nonsteroidal anti-inflammatory drugs (NSAIDs), antibiotics such as cephalosporins, aminopenicillins and rarely macrolides, as shown in the EuroSCAR study, together with the REACT project and the regiSCAR project [17–21].

The ALDEN algorithm represents the gold standard tool in SJS/TEN to identify the culprit drug and to discriminate it from the “innocent” drugs which can be safely administered. It gives to each drug taken by patient a score, ranging from −12 to +10, which corresponds to the probability of having caused the reaction, ranging from very unlikely to very probable [10]. Besides, from a recent analysis a significant statistic for agreement (kappa 0.571) was found between ALDEN score for related-drugs (ALDEN ≥ 4) and lymphocyte transformation test (LTT) results performed after recovery [22].

According to the ALDEN algorithm results, the most likely implicated drug in our case is hydroxychloroquine.

Hydroxychloroquine is an 4-aminoquinoline with a low molecular weight (333,9 Da) [23–26]. It has been used for decades to treat rheumatologic conditions (with a dose of 6 mg/kg) with a general good safety, even though in last years cases of skin reactions, also severe, have increasingly been reported [27].

We searched the Eudravigilance database and found 30 cases of TENs related to the use of hydroxychloroquine [28].

Hydroxychloroquine is being profusely used to treat SARS-CoV-2 infection with an unconventionally high dosage (from 200 mg twice a day to 200 mg three times a day, which is independent of body weight sometimes with a loading dose, due to its high volume of distribution).

Interestingly, some new severe skin manifestation to hydroxychloroquine have also been reported during the treatment of SARS-CoV-2 positive patient [29–31].

In this instance, it is not to be discounted that a rare side effect, such as TEN, occurring with a not commonly associated drug, hydroxychloroquine, could have been favored by the particular immune stimulation induced by the virus SARS-CoV-2 [3]. SJS/TEN, and SCAR more generally, have been classically demonstrated to be associated with viral replication [11, 32–34].

To our knowledge, this is the first case of TEN in a COVID-19 positive patient.

Another salient aspect of the case is the favorable evolution of the patient given that this type of SCAR is typically associated with a bad prognosis [35–37], even more so because the patient displayed all the negative typical prognostic factors also for COVID-19 [38–41], indeed the calculation of the severity-of-illness score for toxic epidermal necrolysis (SCORTEN) in our patient led to an estimated mortality rate of 58.3% (CI 36,6 –77,59) (Table 2) [42, 43].

**Table 2:** Calculation of the Severity-of-Illness Score for Toxic Epidermal Necrolysis (SCORTEN) in our patient

Risk factor	Score		Patient score
	0	1	
Age	< 40 years	> 40 years	1
Associated malignancy*	No	Yes	0
Heart rate (beats/min)	< 120	≥ 120	1
Serum urea (mg/dL) [mmol/L]	≤ 28 [10]	> 28 [10]	1
Detached or compromised body surface	< 10%	≥ 10%	1
Serum bicarbonate (mEq/L)	≥ 20	< 20	0
Serum glucose (mg/dL) [mmol/L]	≤ 250 [14]	> 250 [14]	0
Total			4

More risk factors result in a higher score and a higher mortality rate (%) as follows:

0–1 = 3.2% (CI: 0.1-16.7)

2 = 12.1% (CI: 5.4-22.5)

3 = 35.5% (CI: 19.8-53.5)

4 = 58.3% (CI: 36.6-77.9)

≥5 = 90% (CI: 55.5-99.8)

CI: Confidence Interval [42, 43]

^a^Malignancy: evolving cancer and hematological malignancies

It is possible that the prompt diagnosis of TEN, the suspension of all the suspected drugs (in particularly hydroxychloroquine) and the administration of steroid and IVIG, both targeting the inflammatory syndrome initially triggered by the virus, may have had contributed to a better prognosis, [1, 5].

Further studies are needed to assess the safety profile of hydroxychloroquine in patients with COVID-19.

## Conclusion

To our knowledge, this is the first case of TEN associated with hydroxychloroquine in patient suffering from COVID-19. Given the widespread off-label use of this drug, the high degree of immune activation induced by the virus and the recent increase of hydroxychloroquine-related SCARs, we believe that it is necessary to adequately monitor the skin in all COVID-19 positive patients treated with hydroxychloroquine in order to early detect early signs of toxicities.

## Supplementary information

**Additional file 1:** Details regarding the calculation of the ALDEN score [10].

**Additional file 2:** Timing of the drugs administered to the patients regarding the calculation of the ALDEN score [10].

## Data Availability

Not applicable.

## Abbreviations

CK: Creatinine Kinase
COVID-19: COronaVIrus Disease 19
CTLs: Cytotoxic T Lymphocytes
DRESS: Drug Reaction with Eosinophilia and Systemic Symptoms
HLA: Human Leukocyte Antigen
IVIG: IntraVenous Immunoglobulin
LDH: Lactate Dehydrogenase
LTT: Lymphocyte Transformation Test
MHC: Major Histocompatibility Complex
NK: Natural Killer
NSAIDs: NonSteroidal Anti-Inflammatory Drugs
SARS-CoV-2: Severe Acute Respiratory Syndrome– - Coronavirus—2
SCAR: Severe Cutaneous Adverse Reaction
SCORTEN: Severity-of-illness Score for Toxic Epidermal Necrolysis
SSSS: Staphylococcal Scalded Skin Syndrome
SJS: Stevens Johnson Syndrome
TCR: T-Cell Receptor
TEN: Toxic Epidermal Necrolysis
