# Supplementary material for: A case report of toxic epidermal necrolysis (TEN) in a patient with COVID-19 treated with hydroxychloroquine: are these two partners in crime?
Source: Clin Mol Allergy. 2020 Oct 6;18:19. doi: 10.1186/s12948-020-00133-6 (PMC7537980; doi:10.1186/s12948-020-00133-6)
Supplement: Supplementary file 1 — Additional file 1: Details regarding the calculation of the ALDEN score [10]. [file 12948_2020_133_MOESM1_ESM.docx]

Appendix 1. Details regarding the calculation of the ALDEN score[10]

| DRUG | CRITERION | VALUES | ALDEN SCORE | CAUSAL LINK |
| --- | --- | --- | --- | --- |
| Enoxaparin | Delay from initial drug intake to index day | +3 | -2 | Very unlikely |
|  | Drug present in the body (on index day) | -1 |  |  |
|  | Pre-challenge/Re-challenge | -2 |  |  |
|  | De-challenge | 0 |  |  |
|  | Type of drug (notoriety) | -1 |  |  |
|  | Other cause | -1 |  |  |
| Oseltamivir | Delay from initial drug intake to index day | +3 | -2 | Very unlikely |
|  | Drug present in the body (on index day) | -3 |  |  |
|  | Pre-challenge/Re-challenge | 0 |  |  |
|  | De-challenge | 0 |  |  |
|  | Type of drug (notoriety) | -1 |  |  |
|  | Other cause | -1 |  |  |
| Ceftriaxone | Delay from initial drug intake to index day | +3 | +1 | Unlikely |
|  | Drug present in the body (on index day) | -3 |  |  |
|  | Pre-challenge/Re-challenge | 0 |  |  |
|  | De-challenge | 0 |  |  |
|  | Type of drug (notoriety) | 2 |  |  |
|  | Other cause | -1 |  |  |
| Potassium Canrenoate | Delay from initial drug intake to index day | +3 | -3 | Very Unlikely |
|  | Drug present in the body (on index day) | -3 |  |  |
|  | Pre-challenge/Re-challenge | -2 |  |  |
|  | De-challenge | 0 |  |  |
|  | Type of drug (notoriety) | 0 |  |  |
|  | Other cause | -1 |  |  |
| Pantoprazole | Delay from initial drug intake to index day | +3 | -2 | Very unlikely |
|  | Drug present in the body (on index day) | -3 |  |  |
|  | Pre-challenge/Re-challenge | -2 |  |  |
|  | De-challenge | 0 |  |  |
|  | Type of drug (notoriety) | 1 |  |  |
|  | Other cause | -1 |  |  |
| Hydroxychloroquine | Delay from initial drug intake to index day | +3 | +4 | Possible |
|  | Drug present in the body (on index day) | 0 |  |  |
|  | Pre-challenge/Re-challenge | 0 |  |  |
|  | De-challenge | 0 |  |  |
|  | Type of drug (notoriety) | 1 |  |  |
|  | Other cause | 0 |  |  |
| Piperacillin / Tazobactam | Delay from initial drug intake to index day | +3 | +1 | Unlikely |
|  | Drug present in the body (on index day) | -3 |  |  |
|  | Pre-challenge/Re-challenge | 0 |  |  |
|  | De-challenge | 0 |  |  |
|  | Type of drug (notoriety) | 2 |  |  |
|  | Other cause | -1 |  |  |
| Dexamethasone | Delay from initial drug intake to index day | +3 | 0 | Unlikely |
|  | Drug present in the body (on index day) | -3 |  |  |
|  | Pre-challenge/Re-challenge | 0 |  |  |
|  | De-challenge | 0 |  |  |
|  | Type of drug (notoriety) | 1 |  |  |
|  | Other cause | -1 |  |  |
| Levofloxacin | Delay from initial drug intake to index day | 1 | +1 | Unlikely |
|  | Drug present in the body (on index day) | 0 |  |  |
|  | Pre-challenge/Re-challenge | 0 |  |  |
|  | De-challenge | 0 |  |  |
|  | Type of drug (notoriety) | 1 |  |  |
|  | Other cause | -1 |  |  |
| Paracetamol | Delay from initial drug intake to index day | 3 | -1 | Very Unlikely |
|  | Drug present in the body (on index day) | -3 |  |  |
|  | Pre-challenge/Re-challenge | -2 |  |  |
|  | De-challenge | 0 |  |  |
|  | Type of drug (notoriety) | 2 |  |  |
|  | Other cause | -1 |  |  |
