# Supplementary material for: A case report of toxic epidermal necrolysis (TEN) in a patient with COVID-19 treated with hydroxychloroquine: are these two partners in crime?
Source: Clin Mol Allergy. 2020 Oct 6;18:19. doi: 10.1186/s12948-020-00133-6 (PMC7537980; doi:10.1186/s12948-020-00133-6)

Appendix 2. Timing of the drugs administered to the patients regarding the calculation of the ALDEN score[10]


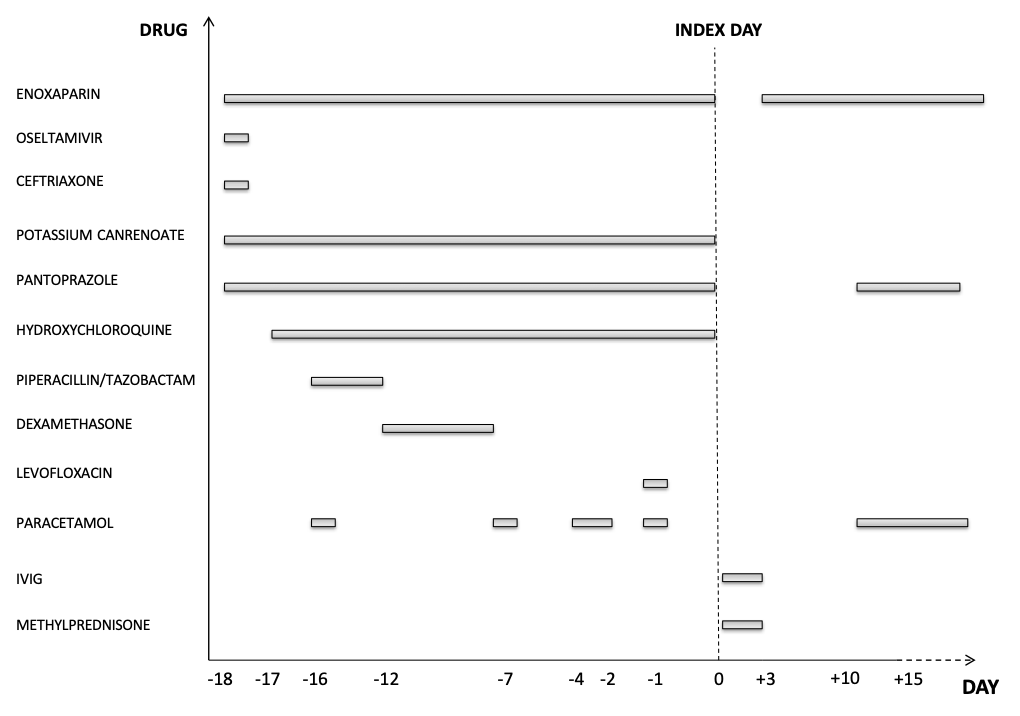

Supplement: Supplementary file 2 — Additional file 2: Timing of the drugs administered to the patients regarding the calculation of the ALDEN score [10]. [file 12948_2020_133_MOESM2_ESM.docx]
